# Giant carotid chemodectoma treated with a combination of surgery and CyberKnife radiotherapy: a case report and review of the literature

**DOI:** 10.1186/s13256-021-03237-y

**Published:** 2022-02-25

**Authors:** José M. López-Arcas, César M. Colmenero, Roberto Martínez, Fátima Martín-Hernán, Beatriz Ruiz-Sánchez, Juan Manuel Aragoneses

**Affiliations:** 1 Oral and Maxillofacial Surgeon. University Hospital Infanta Leonor, Madrid, Spain; 2 Head of the Craniofacial and Maxillofacial Unit, Ruber International Hospital, Madrid, Spain; 3 Chief Cyberknife Radiosurgery Unit. Hospital Ruber Internacional, Madrid, Spain; 4 Alfonso X el Sabio University, Madrid, Spain; 5 Department of Dental Research, Federico Henriquez y Carvajal University, Santo Domingo, 10106 Dominican Republic

**Keywords:** Chemodectoma, Carotid sheath tumors, Radiotherapy, CyberKnife

## Abstract

**Background:**

Paragangliomas are rare vascular neuroendocrine tumors that develop in the extra-adrenal paraganglion tissue. They occur most commonly at the carotid bifurcation, where they are known as carotid body tumors. Most paragangliomas are benign, locally aggressive, infiltrative tumors. Approximately 10% of patients with paragangliomas develop distant metastases, 10% present with multiple or bilateral tumors (mostly carotid body tumors), and 10% have a family history of paragangliomas. The malignant transformation of carotid body tumors has been reported in 6% of cases.

**Case presentation:**

We present the case of a 64 year-old Caucasian woman with a gigantic glomic tumor mass in the neck. Twenty years before the consultation, the patient had undergone an unsuccessful attempt to remove the mass. Over the last 3 years, the patient had felt enlargement of the mass at an increased rate, almost doubling the prior size. Angio magnetic resonance imaging showed a 9 cm paratracheal mass on the left cervical side that laterally displaced the sternocleidomastoid muscle and 2 c m of the trachea. Due to the change in the tumor behavior, the maxillofacial team at Ruber International Hospital decided to remove the tumor surgically after embolization. During the surgery the tumor was gently dissected from the carotid an removed from the carotid bifurcation uneventfully. Two small nodes adhering tightly to the internal carotid adventitia and the posterior torn hole were left in place to avoid any potentially life-threatening complications. The final biopsy confirmed the initial diagnosis of carotid body paraganglioma and showed a Ki-67 expression of 19%. Due to the aggressive growth behavior and high Ki-67 expression of the tumor, the patient was referred to the CyberKnife Unit of Ruber International Hospital for treatment of the remaining nodes.

**Conclusions:**

The management of cervical paragangliomas is difficult and remains a challenge. Although the likelihood of tumor control is high with surgical or radiotherapeutic treatments, we currently lack consensus regarding the best treatment option. Nevertheless, in selected complex cases, such as the case we present, the combination of surgery and radiosurgery may allow complete local tumor control with minimal morbidity.

## Background

Paragangliomas are rare vascular neuroendocrine tumors that develop in the extra-adrenal paraganglion tissue. They represent less than 0.5% of all head and neck tumors. In the head and neck, paragangliomas are named after their site of origin. They occur most commonly at the carotid bifurcation, where they are known as carotid body tumors. Additional sites of origin include the jugular bulb (jugular paraganglioma), the vagus nerve (vagal paraganglioma), and within the middle ear mucosa (tympanic paraganglioma) [1].

These tumors grow slowly, and they are reported to have a median doubling time of 4.2 years. Without therapy, they may grow to a considerable size and become life threatening.

Most paragangliomas are benign, locally aggressive, infiltrative tumors. Approximately 10% of patients with paragangliomas develop distant metastases, 10% present with multiple or bilateral tumors (mostly carotid body tumors), and 10% have a family history of paragangliomas. The malignant transformation of carotid body tumors has been reported in 6% of cases [1–6].

The clinical signs and symptoms of benign and malignant paragangliomas overlap significantly. Most lesions produce local compressive symptoms including hearing loss, otalgia, tinnitus, neck mass, or cranial neuropathy. Cranial neuropathy can lead to dysphagia, facial weakness, and vocal fold paresis. A small proportion of head and neck paragangliomas may also show biochemical activity similar to that of pheochromocytomas, possibly inducing symptoms of diarrhea, hypertension, and flushing [4].

The diagnosis of paragangliomas is generally based on radiological appearance because biopsy is challenging due to the anatomical location and vascularity. The literature contains little information about the specific clinical behavior of malignant paragangliomas that would help to distinguish them from benign lesions prior to therapy. Furthermore, the diagnosis of malignant paraganglioma is problematic and often relies on final pathological results [7–10].

Several of these tumors are inherited through mutations of the genes that encode for the succinate dehydrogenase enzymes SDHD and SDHB. Although the inheritance of both genes is autosomal dominant, the SDHD gene is subject to maternal imprinting, and the SDHB gene has incomplete penetrance. Patients with the inherited gene almost invariably have multiple tumors [11, 12].

Three treatment options are currently recognized: surgical resection, radiation therapy, and a wait-and-scan policy. Of these, surgery is the only curative treatment. However, because surgery may be complicated by significant morbidity, especially in larger tumors, it is considered by some authors to be controversial.

Here, we present the case of a giant paraganglioma body tumor treated with a combined surgical and radiotherapeutic (CyberKnife) protocol. This case report review has been approved by the Ethics Committee at the Ruber International Hospital.

## Case presentation

**Fig. 1:**
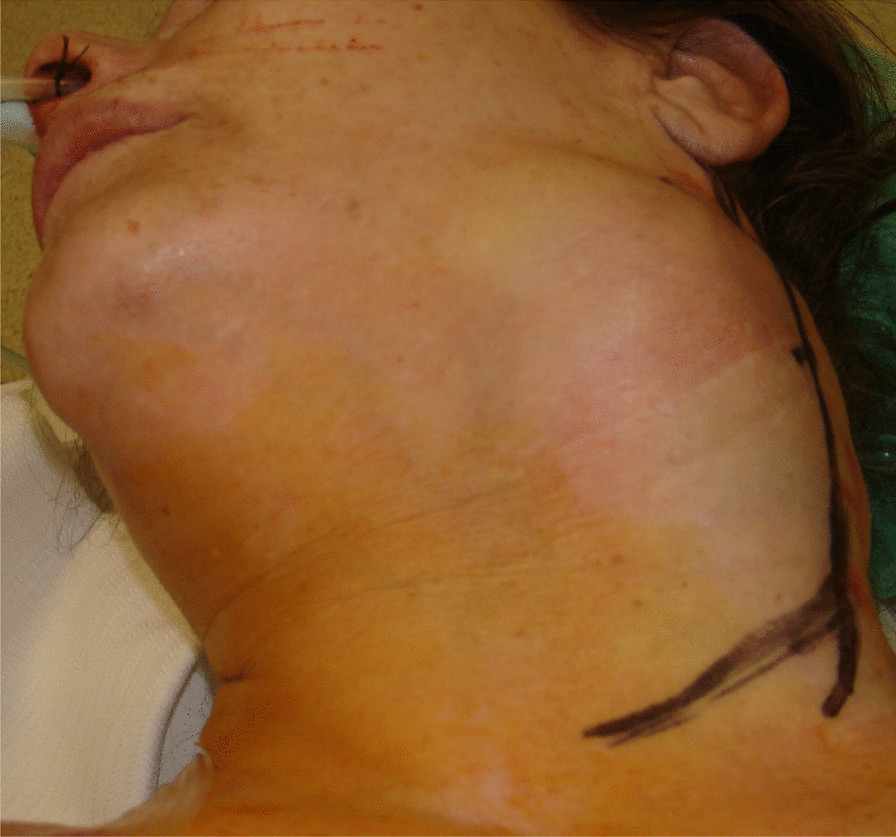
Preoperative image shows the large mass protruding in the left side of the neck

A 64-year-old Caucasian woman was referred to our office for the assessment of a gigantic left neck mass (Fig. 1). The patient reported that she had started to feel a paratracheal node 34 years previously, and that it had grown progressively during the subsequent three decades. She had undergone an operation in the area 24 years previously, although the surgery had to be terminated due to massive bleeding from the mass. During surgery, the mass was diagnosed as most likely a glomus tumor. No further surgeries to remove the mass were attempted, and it had been allowed to grow steadily.

In May 2013, the patient was referred elsewhere for further study of the mass. Magnetic resonance imaging (MRI) revealed a hypervascularized mass with a maximum diameter of 4 cm located at the bifurcation of the left carotid artery and causing the forward displacement of the external carotid artery and the backward displacement of the internal carotid artery. The patient refused surgery at that time and remained asymptomatic for almost 3 years. She then decided to consult a physician again due to the enlargement of the mass, which caused discomfort and mild compression symptoms when swallowing.

**Fig. 2:**
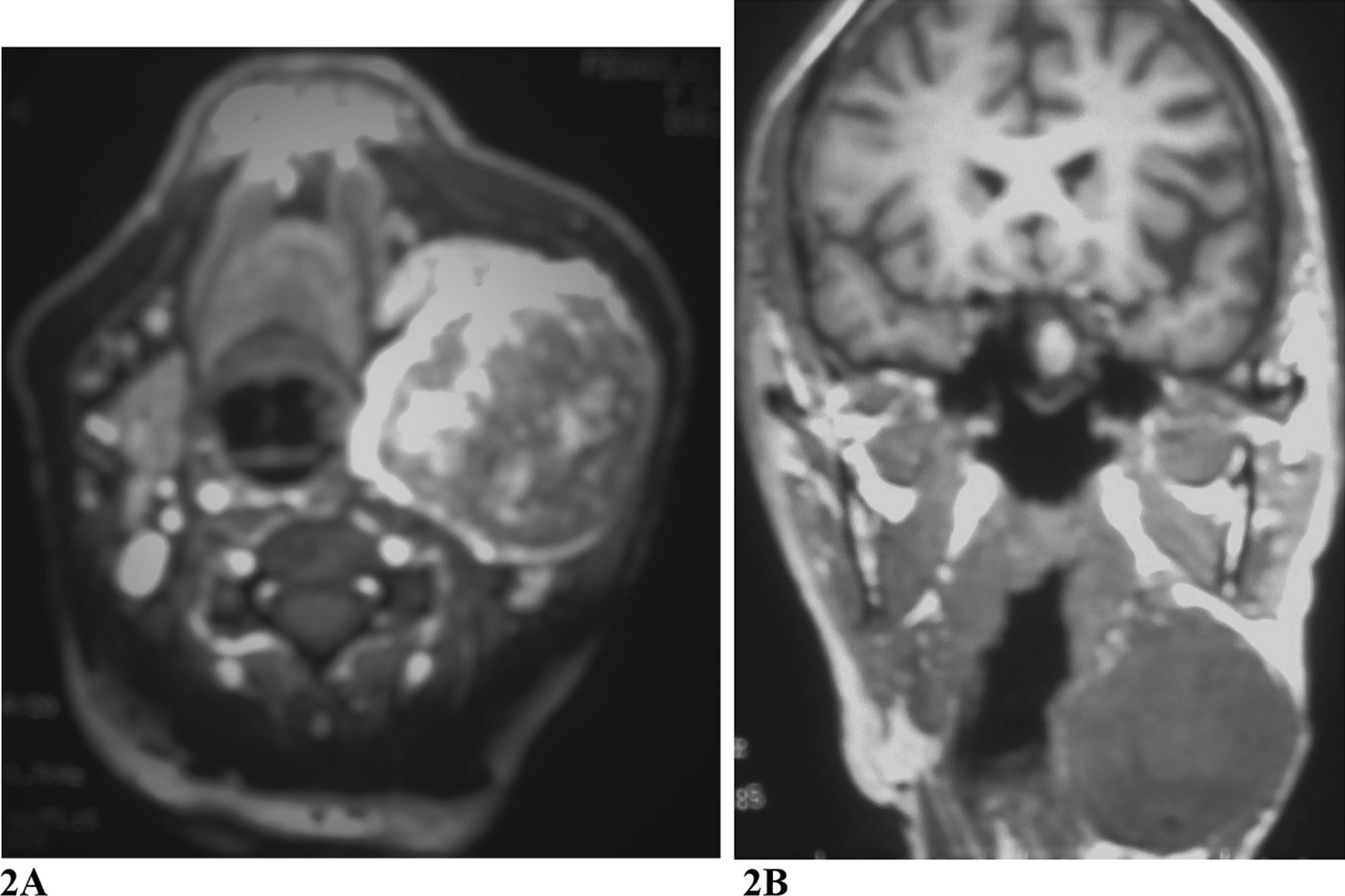
**A** Angio MRI image showed a 9 cm paratracheal mass on the left cervical side that laterally displaced the sternocleidomastoid muscle and 2 cm to the right side the trachea. **B** Coronal axial vascular MRI showing the same paratracheal mass on the left cervical side involving the carotid bifurcation

In 2016, angio MRI (Fig. 2) showed a 9 cm paratracheal mass on the left cervical side that laterally displaced the sternocleidomastoid muscle and 2 cm of the trachea. The mass was pulsatile and slightly painful. No Horner syndrome or Hering–Breuer reflex was noted. A carotid occlusion test and a complete angiography were also performed, and these confirmed the permeability of the Willis polygon and the diagnosis of the mass.

Due to the change in the behavior of the tumor, which had doubled in size within 3 years after remaining relatively stable for almost three decades, the size of the tumor, which had started causing compression symptoms, and the Shamblin grade II classification, the maxillofacial team at Ruber International Hospital decided to remove the tumor surgically after embolization.

**Fig. 3:**
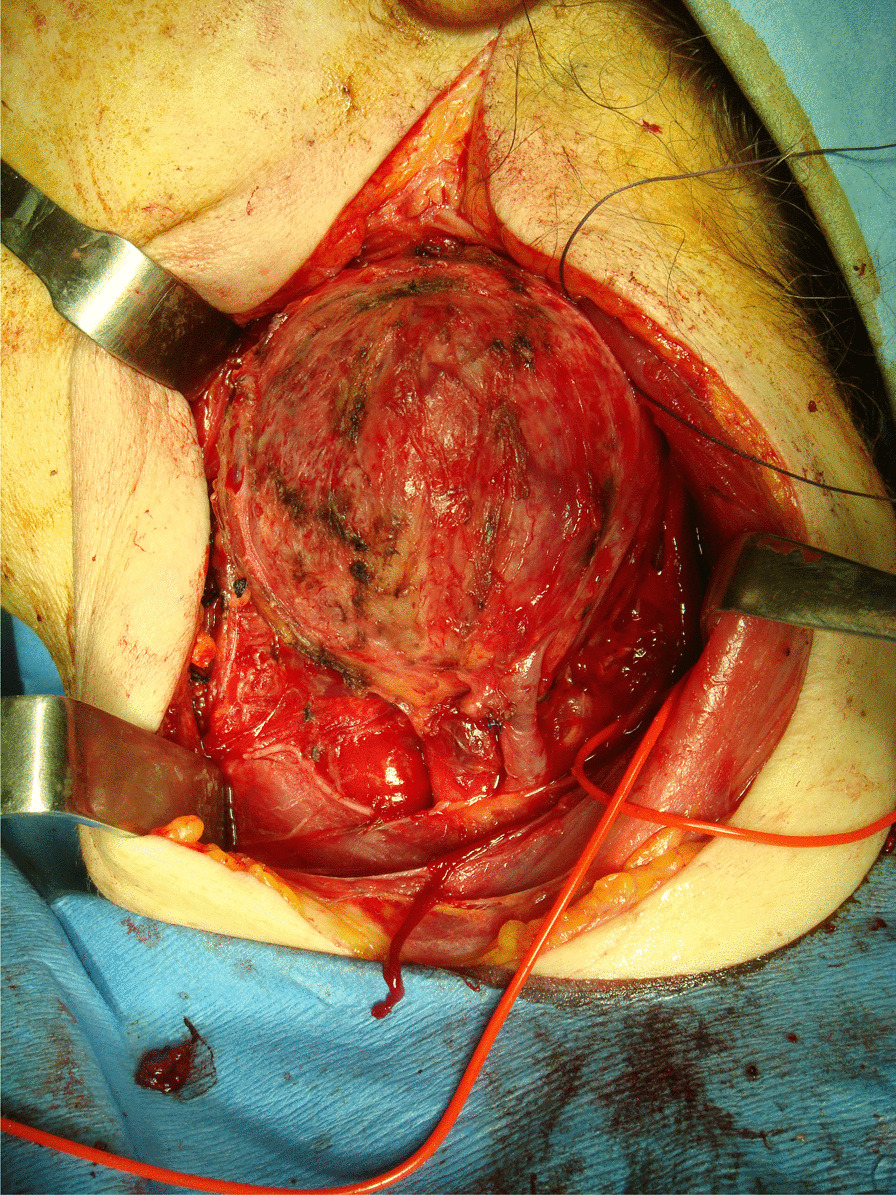
Neck mass appearance at the beginning of the dissection

The surgery was performed in February 2016. The tumor was approached through a wide cervical exposure after identifying the primitive carotid artery (Fig. 3).

**Fig. 4:**
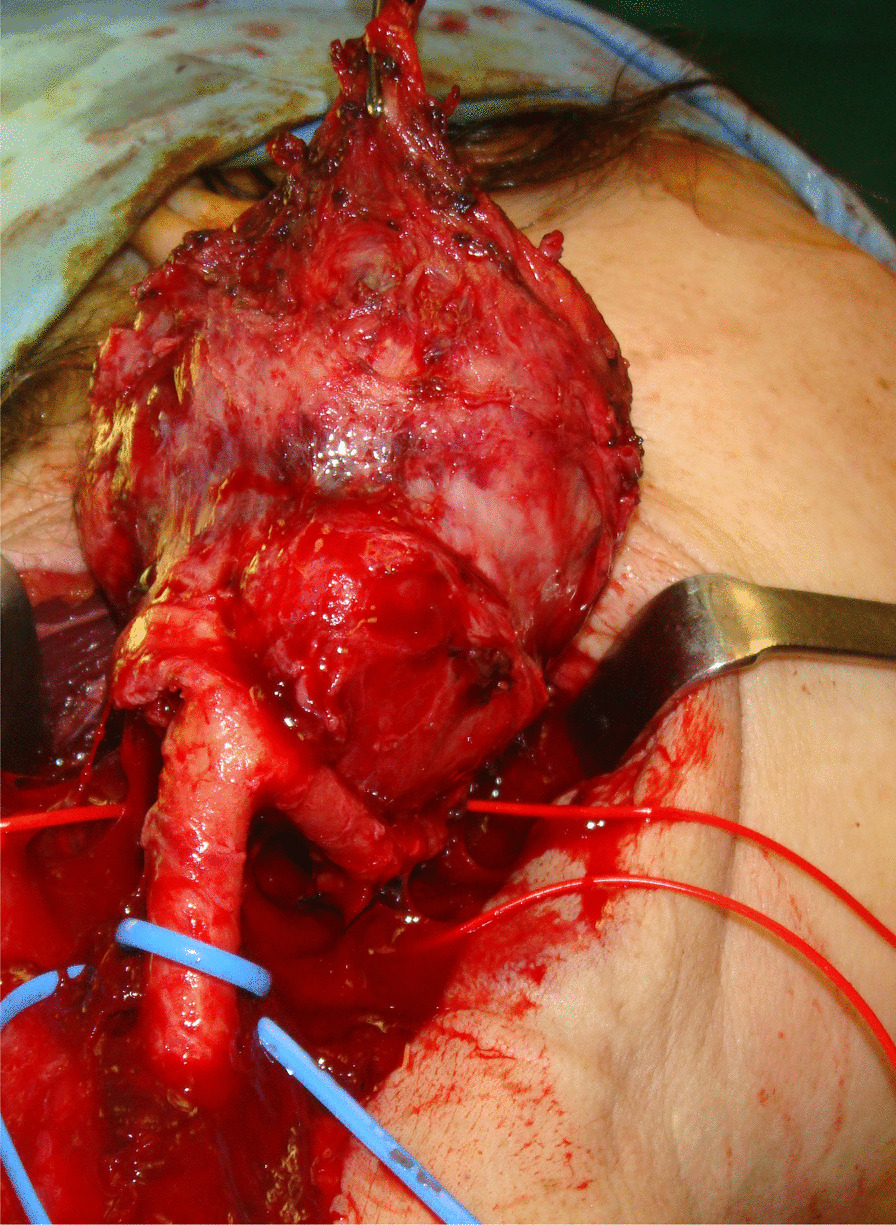
Paraganglioma showing its relationship to the carotid glomus

The tumor was gently dissected from the carotid bifurcation in a subadventitial plane (Fig. 4), with great emphasis placed on maintaining hemostasis.

**Fig. 5:**
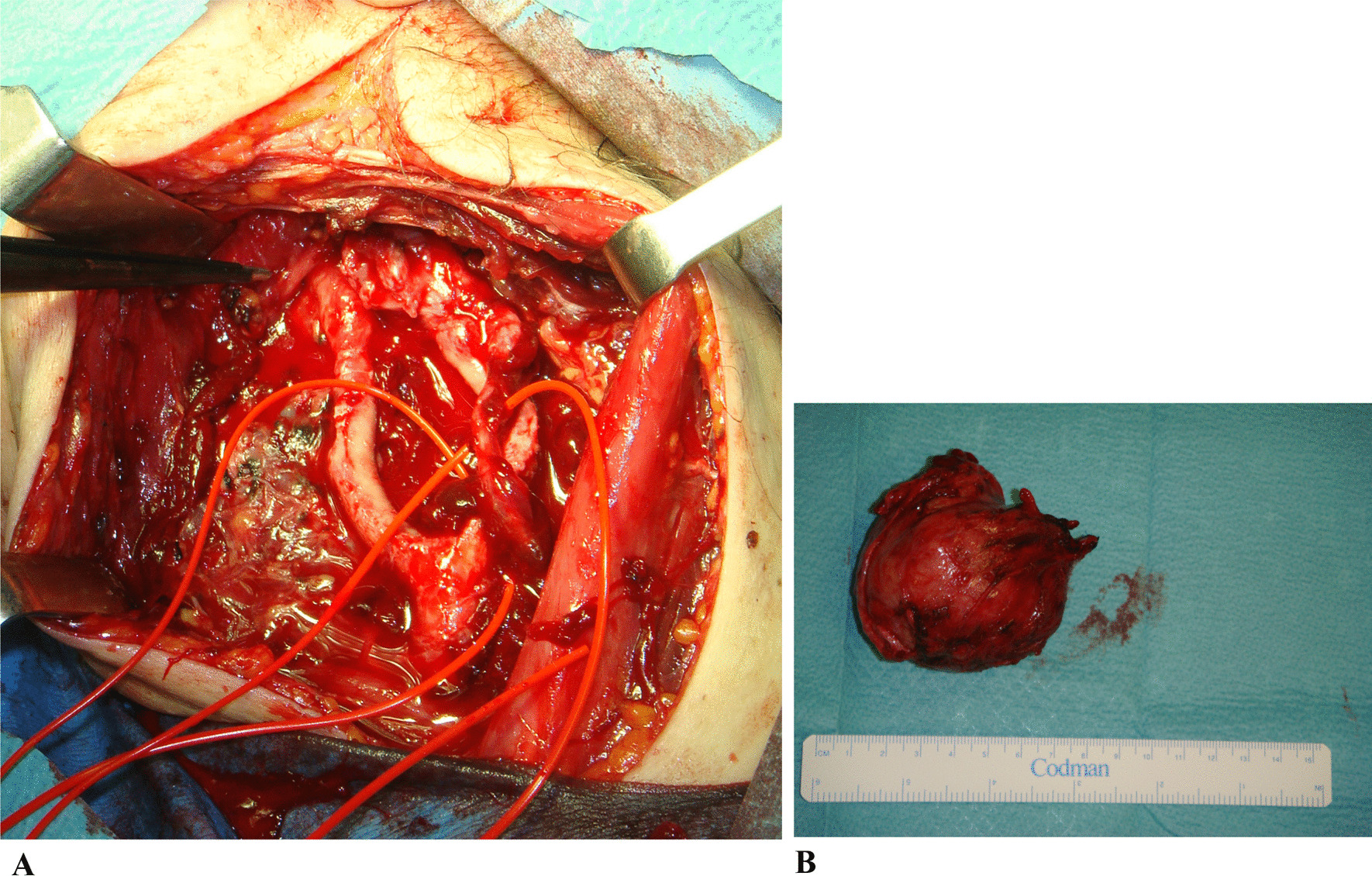
**A** Surgical field following paraganglioma removal. The great displacement of the internal and the external carotid arteries at the glomus due to the progressive enlarging mass protrusion of the paraganglioma can be noted. **B** Surgical specimen

The surgeons would like to note that a partially blunt Freer periostotome is the best tool to use in high-risk areas. The vagal nerve was readily identified and separated from the mass. The hypoglossal nerve and major neck vessels were also preserved (Fig. 5A, B). The mass was then removed from the bifurcation uneventfully. Two small nodes adhering tightly to the internal carotid adventitia and the posterior torn hole were left in place to avoid any potentially life-threatening complication.

The postoperative phase was uneventful, and the patient was discharged 6 days after the surgery with moderate hoarseness, which was resolved within 1 month. The final biopsy confirmed the initial diagnosis of carotid body paraganglioma and showed a Ki-67 expression of 19%.

**Fig. 6:**
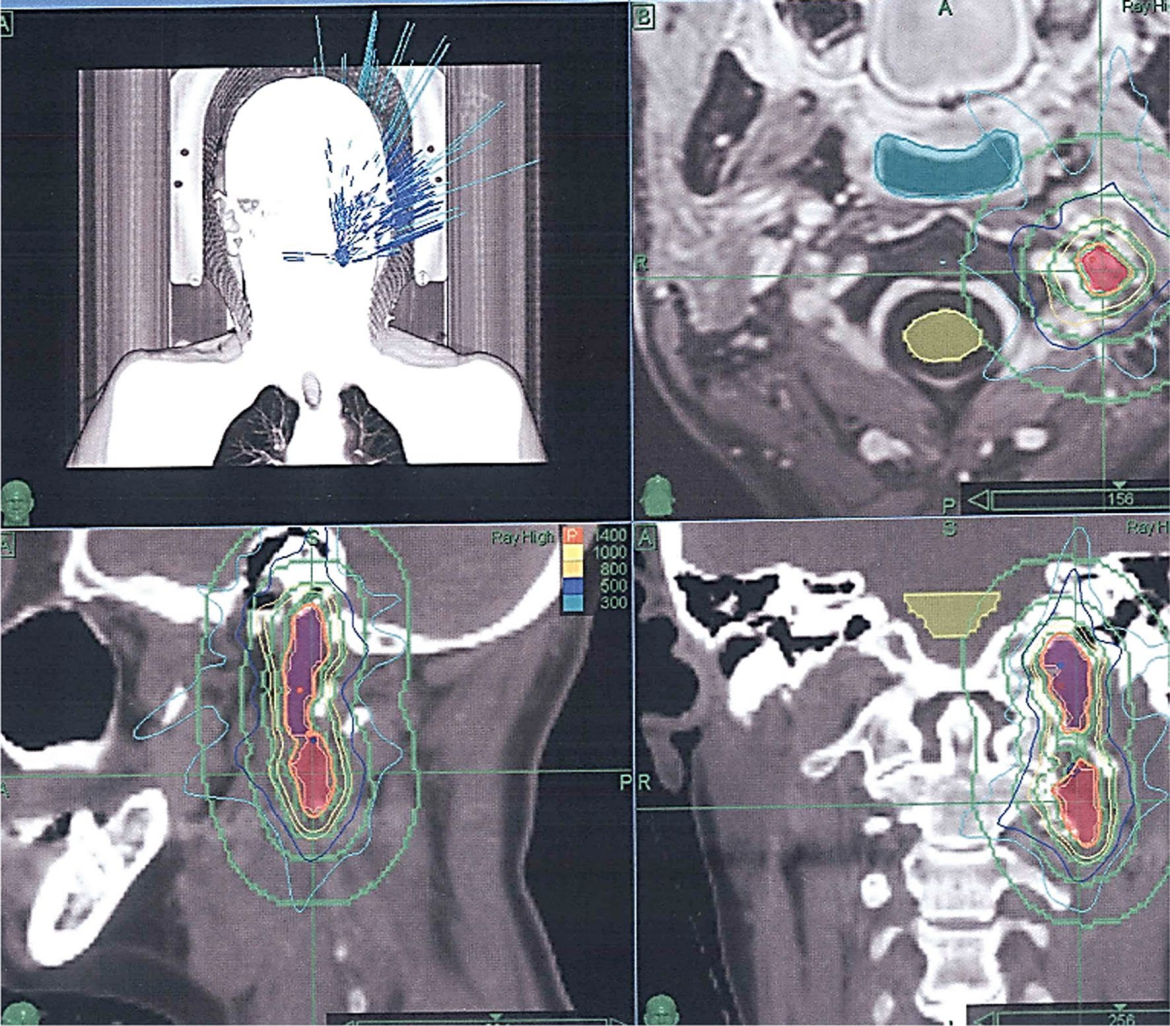
CyberKnife planning: Coverage dose of 14 Gy and an isodose of 83% were administered using 5- and 7.5-mm collimators. The maximum dose used was 16.87 Gy. The medullary canal received a dose of less than 4 Gy, and the left VII and VIII nerves received a dose of less than 5 Gy

Due to the aggressive growth behavior and high Ki-67 expression of the tumor, the patient was referred to the CyberKnife Unit of Ruber International Hospital for treatment of the remaining nodes. A thermoplastic mask was made to ensure that the patient remained immobilized during the treatment. A T2 MRI and computed tomography (CT) with intravenous contrast were performed to locate the above-mentioned remaining nodes of the paraganglioma. The treatment was performed separately in each affected area on two consecutive days. A coverage dose of 14 Gy and an isodose of 83% were administered using 5- and 7.5-mm collimators. The maximum dose used was 16.87 Gy. The medullary canal received a dose of less than 4 Gy, and the left VII and VIII nerves received a dose of less than 5 Gy (Fig. 6). The treatment course was uneventful, and no complication occurred during or after treatment.

**Fig. 7:**
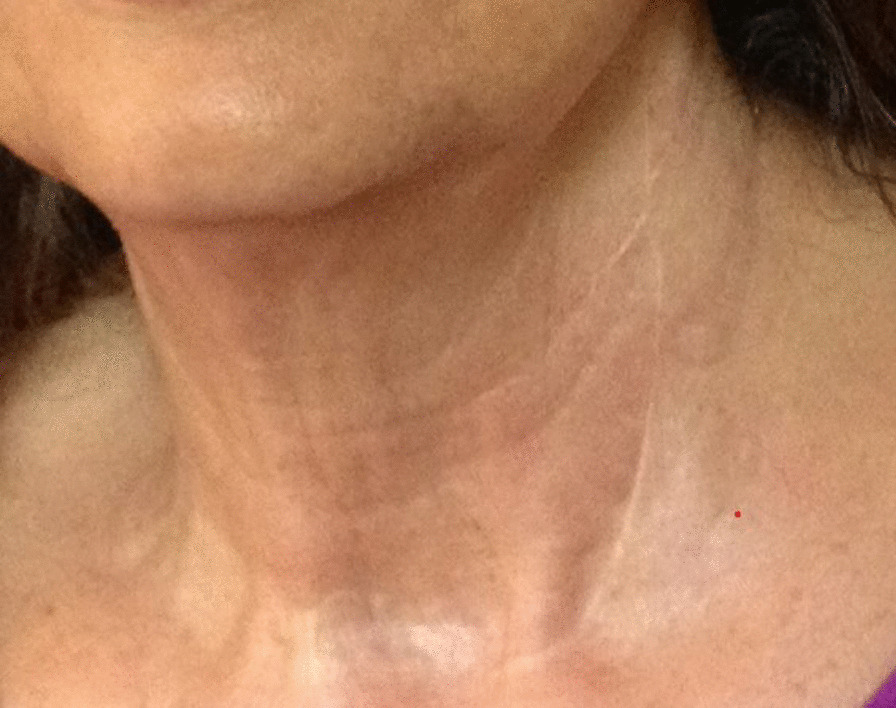
Two-year postoperative result. No recurrence has been detected

After a 48-month follow-up period, no lesion recurrence or surgery-related complications have occurred. Some minor scar revision due to the tracheostomy was performed under local anesthesia. The patient is otherwise completely symptom free (Fig. 7).

## Discussion and conclusions

The carotid body tumor is the most common type of paraganglioma in the head and neck, and is the only pathology to affect the carotid body [1–3]. Carotid body tumors may be sporadic or familial; the sporadic form is slightly more common in women [3, 10, 11]. These tumors often present as slow-growing, non-tender neck masses located just anterior to the sternocleidomastoid muscle at the level of the thyroid [4].

The differential diagnosis includes cervical lymphadenopathy, carotid artery aneurysm, brachial cleft cyst, laryngeal carcinoma, and metastatic tumor. Carotid body tumors are typically mobile in the lateral plane but have restricted mobility in the cephalocaudal direction (Fontaine sign). Occasionally, such tumors may transmit the carotid pulse or demonstrate a bruit or thrill. Due to the close proximity to the carotid vessels and cranial nerves X–XII, tumor enlargement may cause progressive neurological symptoms, such as dysphagia, odynophagia, or hoarseness [4–6].

Histologically, carotid body tumors resemble the normal architecture of the carotid body. The tumors are highly vascular and contain clusters of tumor cells between the capillaries, producing the pseudoalveolar pattern of Zellballen (cell balls) [1, 2, 7, 8]. These cells have fine-grained eosinophilic cytoplasm and small round or oval nuclei. The malignant potential of these tumors cannot be predicted by histological studies. The diagnosis of malignancy is based on the invasion of lymph nodes, vessels, nerves, the airway, or the base of the skull, rather than on tumor size or histological features [9]. Chapman *et al.* [4] recently suggested that pain, a rapidly enlarging neck mass, and younger age are predictive factors of underlying malignancy, which should prompt the consideration of an aggressive diagnostic and management approach.

Contrast CT and MRI [9] have evolved as effective noninvasive imaging modalities for the detection of carotid body tumors. They commonly demonstrate tumor blood supply and the widening of the carotid bifurcation by a well-defined tumor blush (Lyre sign), which is a classic pathognomonic contrast angiographic finding. Carotid angiography also plays an important role in the evaluation and management of these tumors. Angiography demonstrates the vascular extent of the tumor, carotid artery involvement, the size and location of tumor-feeding vessels (useful for evaluating the possibility of tumor embolization), and coexisting atherosclerotic disease of the carotid arteries.

The management of carotid body tumors has been controversial because of their low malignant potential and high rates of operative complications. Early experiences with carotid body tumor resections were associated with operative mortality rates ranging from 5% to 13%, postoperative cranial nerve palsy in 32–44% of patients, and other postoperative neurological complications in 8–20% of patients. However, most recent experiences have demonstrated the safety of the surgical approach, with mortality rates of 1–2% but a high (40%) morbidity rate due to cranial nerve injury and cerebral ischemic events [12]. Surgical treatment has been the standard approach to symptomatic carotid body tumors for many years and has shown high cure rates [12].

The size of a carotid body tumor is a very important consideration in the development of a treatment strategy. As in the case reported here, tumors larger than 4–5 cm tend to show partial or complete encirclement of the carotid arteries. Larger tumors have been associated with higher risks of bleeding and cranial nerve injury during surgery. Preoperative tumor embolization has been employed to reduce tumor size and thereby decrease the risk of complications. The Shamblin classification system (Table 1), which is based on tumor size, was proposed in 1971. According to this classification, group I tumors are relatively small and minimally attached to carotid vessels; the surgical excision of these tumors is not difficult. Group II tumors, including the case reported here, are large and show moderate attachment to the carotid vessels; these tumors are amenable to careful surgical excision. Group III tumors are very large and encase the carotid vessels; these tumors often require arterial resection and grafting [13].

**Table 1:** Shamblin classification of carotid body paraganglioma. Shamblin, 1971 [13]

Group	Features
I	Tumors < 4 cm in size not surrounding or infiltrating the carotid and excision done without difficulty
II	Tumors > 4 cm in size partially surrounding or infiltrating the carotid and excision done with difficulty
IIIa	I, II or III infiltration of carotid vessel; > 4 cm in size and intimately infiltrating or surrounding the carotid vessels with difficulty requiring vascular repair, sacrifice, or vessel replacement
IIIb	

As previously described, a thorough subadventitial dissection with proximal control of the primitive carotid artery and the early identification and dissection of the cranial nerves (mainly the vagal and hypoglossal nerves) help to prevent much of the morbidity related to the surgical excision.

Recent studies have reported on the use of radiotherapy as the first-choice treatment for paragangliomas [14]. The main arguments in favor of this treatment are that it is less invasive than surgery, has fewer complications, and achieves high rates of local control. Hinerman and colleagues [14] reported a 96–100% tumor control rate in cervical paragangliomas after radiation therapy using a dose of 45 Gy. However, the cure criterion for radiotherapy, which is growth cessation rather than disappearance, is difficult to evaluate [15]. Thus, in gigantic tumors such as the one presented here, radiotherapy may stop growth but will not reduce the tumor mass. In such cases, compression symptoms may not worsen but will not disappear.

Further complications of radiation therapy may include inflammation of the external auditory canal and middle ear, osteoradionecrosis, cranial nerve neuropathies, and direct injury to brain tissue. Furthermore, it must be remembered that surgery will be more difficult if radiation therapy fails [12].

Histological examination has shown that the chief cells are minimally affected by radiation; rather, the distinctive vascular structure of the tumor is replaced by fibrous connective tissue [15, 16]. This development is of concern in potentially malignant tumors, which have been reported in 3–5% of cases. In our case, we used the predictive factors of Chapman *et al.* [4] to consider the possibility of malignant transformation of the chemodectoma. Given the wide experience of the surgical team with this type of tumor, surgical excision was selected as the primary treatment option.

Radiosurgery has arisen as a promising approach to the management of paragangliomas, especially glomus jugular tumors. Radiosurgery provides a high degree of accuracy, exquisite precision, and rapid radiation-dose falloff at the peripheries of the target lesions, allowing the clinician to deliver a high radiation dose to neoplastic tissue while sparing healthy brain tissue. This treatment is also relatively noninvasive and can be performed as an outpatient procedure [17–21].

In the case reported here, a preoperative goal of zero morbidity was defined according to the patient’s desires. Therefore, although the tumor resection was practically completed, we did not attempt to remove the nodes adhering most strongly to the skull base and internal carotid artery to avoid taking any further risks. Thus, gamma-knife radiosurgery was crucial to ensure effective local disease control with minimal morbidity.

In summary, the management of cervical paragangliomas is difficult and remains a challenge. Treatment must be individualized, taking into account the patient’s age, tumor site and size, multicentricity, and preexisting cranial nerve deficits. Although the likelihood of tumor control is high with surgical or radiotherapeutic treatments, we currently lack consensus regarding the best treatment option. Nevertheless, in selected complex cases, the combination of surgery and radiosurgery may allow complete local tumor control with minimal morbidity.
